# Submicrometer Microelectronics Dimensional Metrology: Scanning Electron Microscopy

**DOI:** 10.6028/jres.092.018

**Published:** 1987-06-01

**Authors:** Michael T. Postek, David C. Joy

**Affiliations:** National Bureau of Standards Gaithersburg, MD 20899; AT&T Bell Laboratories Murray Hill, NJ 07974

**Keywords:** critical dimension, dimension, linewidth, measurements, metrology, micrometer, SEM, standards, submicrometer

## Abstract

The increasing integration of microelectronics into the submicrometer region for VHSIC and VLSI applications necessitates the examination of these structures both for linewidth measurement and defect inspection by systems other than the optical microscope. The low beam-voltage scanning electron microscope has been recently employed in this work due to its potentially high spatial resolution and large depth of field. This paper discusses applications of the scanning electron microscope to microelectronics inspection and metrology in light of the present instrument specifications and capabilities, and relates the scanning electron microscope to the controls required for submicrometer processing.

## Introduction

**Figure 1:**
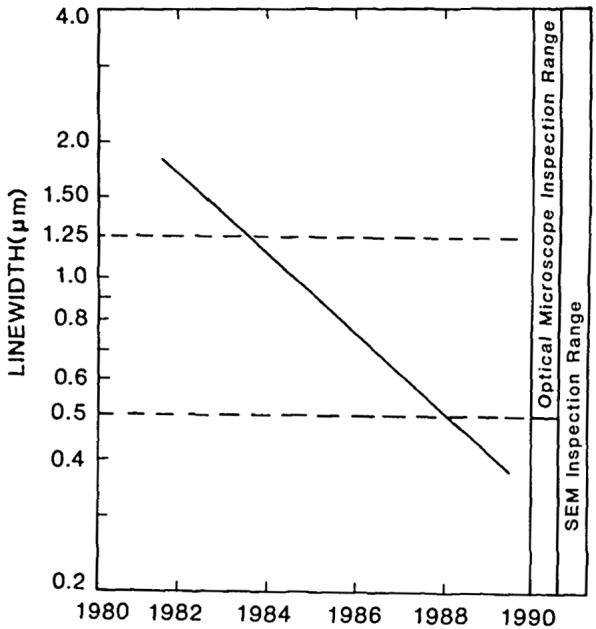
Projected decrease in the size of the linewidth of VHSIC and VLSI circuits through the 1980s and the relationship to optical and scanning electron microscope inspection instrumentation.

The scanning electron microscope (SEM) has become an important tool in the inspection and measurement of microelectronics for the Very Large Scale Integration (VLSI) and Very High Speed Integrated Circuit (VHSIC) programs. As the feature dimensions on integrated circuits reach into the submicrometer region (fig. 1), inspection techniques using scanning electron microscopes are becoming commonplace. Many processing facilities are presently working at a 10%, or even 5% tolerance, in order to produce the precise structures needed for submicrometer circuits. The effect on the process precision of the linewidth measurement is shown in table 1. Application of the “gauge maker’s rule” to the necessary tolerances means that soon the goal for process precision will be in the nanometer range. Even though optical microscopes can be useful for critical linewidth measurement and inspection to about 0.3 μm [1],^1^ many fabrication lines, in anticipation of future needs, are integrating SEMs into the production sequence at chip levels of 1.25 μm geometry and smaller (table 2). Advanced scanning electron beam instruments are presently being developed to facilitate this work and to do automated linewidth measurement and inspection [2–4].

Use of the scanning electron microscope for semiconductor device inspection has several advantages over optical microscopy (table 3), the major advantage being the increased potential resolution due to the much shorter wavelength of the electrons and thus, the ability to circumvent the diffraction effects prevalent in the optical microscope. But, as with anything good, there are also limitations and compromises that complicate the choice.

**Figure 2:**
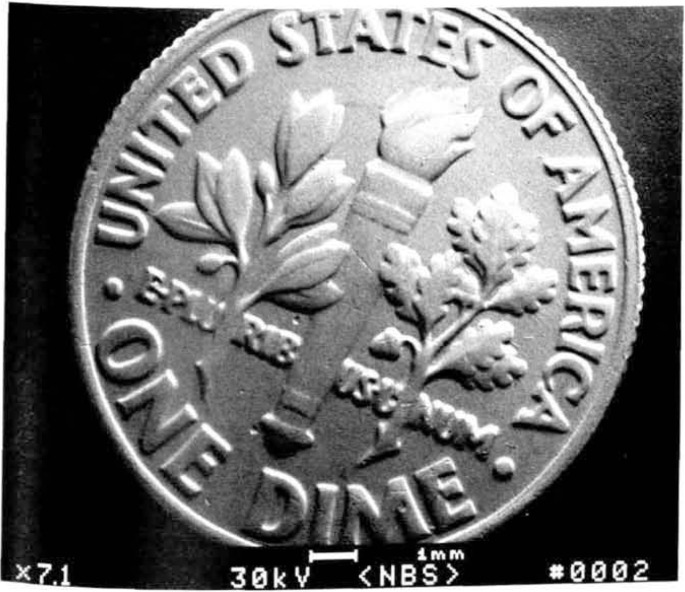
Scanning electron micrograph of a dime demonstrating the importance of proper SEM calibration procedures. Note that the magnification is displayed in the lower left corner and the accelerating voltage displayed in the center. Sec text for full explanation.

The scanning electron microscope is often thought of as a panacea for the measurement needs of the semiconductor community. This is not true today for *accurate* linewidth measurement, but it may ultimately fill that niche as the instrument matures. Unlike the optical microscope which traces its history back to the 1600s and in which optical theory has had a great deal of time to mature, the SEM has only been on the scene as a production instrument since the early-to-mid 1960s and electron optical theory presently is limited by this infancy. The SEM was not originally developed to do the very precise critical dimension measurement required today by the semiconductor manufacturing industry, but as an analytical and picture taking instrument. The mystique surrounding the SEM found its way into semiconductor manufacturing via this route and soon SEM-based measurement followed. In this transition, an attitude developed and was fostered that anything photographed in an SEM was correct. Since the SEM is considered the ultimate authority, measurements made using this instrument are also thought to be indisputably correct. Figure 2 demonstrates a scanning electron micrograph of a common object that everyone should immediately recognize and be able to measure. The magnification is indicated in the lower left corner of the micrograph and, in the center, a line scale or micrometer marker indicates the size of the structure as scaled to the magnification. If the philosophy that everything that comes from a scanning electron microscope is correct, then so is that micrograph. This *could* be typical of any micrograph obtained in a standard instrument. This micrograph seems correct as it resides in the frame of reference of the reader (a dime is small; it easily fits in a pocket, so 7.1× seems proper) therefore, a rough measurement based on the information provided on the micrograph would set the size of the dime as being about 12 mm in diameter. This measurement is smaller than the actual size, for a dime is about 18 mm in diameter. The actual magnification displayed on the micrograph should be about 4.6×. Because it seems reasonable to the reader, the magnification of 7.1× is acceptable. Many micrographs taken of micrometer and submicrometer structures in fabrication facilities also seem reasonable (a submicrometer line is small so such a measurement seems correct) but that does not make them accurate. One has no real firsthand experience in this microscopic world and thus most anything can seem reasonable given the right circumstances. This *specially prepared* micrograph of the dime is designed to prove a point, which is that the SEM does not always tell the truth. The scanning electron-beam instrument *as with any instrument being used for metrology*, will only provide correct data to the observer if it is adjusted to a proper calibration standard, its limitations are understood and strict controls are established and maintained. Without these controls, precise measurements using the SEM are impossible. The engineer, using the SEM to control a process, must look as critically at the micrographs obtained as we now look at the previous figures and he must also ask specific questions of the operator to ensure that the data obtained are really significant and accurate.

For the purpose of discussing SEM metrology, a clear distinction between the terms precision and accuracy must be made at the onset. This is necessary because in many instances these two terms have been erroneously used and treated as if they were synonymous.

### Precision and Accuracy

In metrology [5] the term precision, often referred to as repeatability, is defined as the spread in values associated with the repeated measurements on a given sample using the same instrument under the same conditions. The assumption is that the number of measurements is large, the sample is stable over time and that the errors introduced are random. This is essentially a measure of the repeatability of the instrumentation. Precision relates directly to at least four distinct factors: 1) instrument; 2) operator; 3) environment, and 4) sample. Many of the factors affecting SEM measurement precision will be discussed in later sections of this paper. In order to measure precision, it is not necessary to use an official standard. It is only necessary to use a sample that is of good quality and stable with time. This provides a measure of precision that is locally traceable, and is related to that particular instrument and sample. Furthermore, in the SEM, due to the higher inherent resolution attainable, this precision may only relate to a given section or area of that sample because a sample may vary from location to location. Due to the need for stability with time, the sample materials chosen for these samples may not be identical to the typical product sample of interest (i.e., photoresist). To compare precision between more than one site or instrument would require the particular sample to be carefully transported to the other location and then the test repeated. An adjunct to this would be that an organization (such as NBS) make up and test (with a single instrument) a series of precision test samples which then could be taken to the various sites of interest and the sample precision of the instruments at those sites tested and compared with the measurements made on the original instrument.

Accuracy, on the other hand, is a far more ambiguous concept usually relating to the measurement of some agreed upon quantity (or quantities). Accuracy for SEM metrology is one goal of the program at NBS. This goal is not necessarily identical in principle, or practice, to the goals of the present semiconductor industry, but the results are the same. That is, the production of an accurate SEM standard that can be used to determine the accuracy of semiconductor product measurements. Not only must the above factors affecting precision be considered as limitations of measurement accuracy but also the manner by which a given structure is being measured. Thus, a program similar to that employed for the NBS optical microscope linewidth mask standard must also be undertaken [1]·This program utilizes computer modeling of the electron beam/sample interactions in order to obtain the necessary measurement accuracy. Many of those factors necessary to effectively model linewidth measurements in the SEM are not fully understood at this time [6] and approaches are being developed to quantify them [7,8].

In practice, accuracy may be achieved only if the instrument making the measurement is sufficiently precise and the specimen of interest exactly matches the standard in all important ways (materials, substrate, etc.) except the dimension or dimensions being measured. One complication for linewidth metrology of thick lines (i.e., photoresist, etc.) on wafers is that, even if an acceptable standard were available composed of one set of particular materials, there is no guarantee that a given production sample will match precisely the characteristics of the standard. This is especially true because of the vast number of possible combinations of substrate and resist being used in semiconductor technology today. What may become feasible is the development of an accurate linewidth standard of well established geometry and the parallel development of a computer program to handle the sample and instrumental differences between this standard and the product being measured. This problem is similar in concept to that required for the development of the Z, A, F factors for quantitative x-ray microanalysis and the programs developed at NBS (and other laboratories) to undertake this problem [9]. A program to undertake this challenge is also being implemented.

## The Scanning Electron Microscope Metrology Instrument

**Figure 3:**
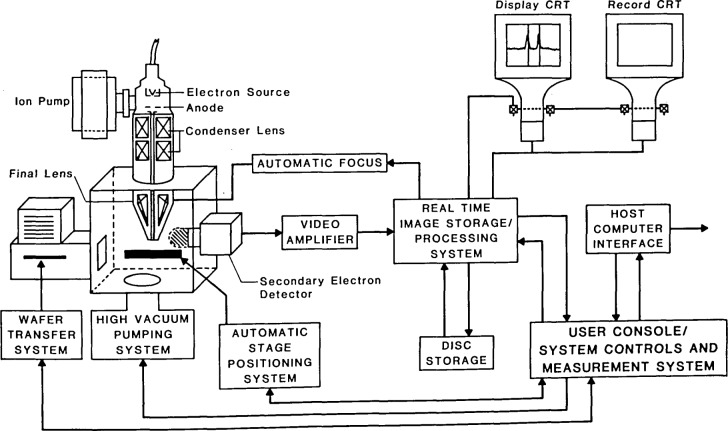
Schematic of a typical scanning electron microscope based wafer inspection instrument. The electron source and column design will vary with manufacture.

The architecture of a typical scanning electron microscope wafer inspection instrument is similar to any modern SEM designed for low accelerating voltage operation with the exception that it is modified to accept and view large semiconductor wafers. The instrument may also have cassette to cassette capabilities to facilitate wafer loading and unloading and a computer-based video profile analysis or “linewidth” measurement system. An example of a generalized instrument is shown in figure 3. In this instrument, a finely focused beam of electrons is moved, or scanned, from point to point on the specimen surface in a precise rectangular motion called a raster pattern. The electrons originate from a filament that may either be heated to a high temperature (thermionic emission), extracted at room or near room temperature (cold field emission) or a combination of both (thermally assisted field emission). Table 4 compares the operational characteristics of the different electron sources presently in use in instruments designed for wafer inspection. The electron gun is the “heart” of the SEM and the overall performance of the instrument ultimately relates to the current density of electrons emitted from the source. The larger this density the better the signal-to-noise ratio and hence the higher the limiting resolution. One measure of the performance characteristics of the electron gun is the measure of brightness (*β*). Brightness is the current density of the electron beam per unit solid angle and is defined by the following:

$$\beta =\frac{4i}{\pi^{2}d^{2}\alpha^{2}}$$ (1)

where *i* is the beam current; *d* is the diameter of the electron beam and *α* is the beam divergence (all measured at the specimen). Brightness is proportional to the current density of the source and it also increases linearly with accelerating voltage [9]. The electron beam, once generated, travels down the column where it undergoes a multistep demagnification with magnetic lenses so that when it impinges on the sample, the beam diameter can range between about 1 nm and 1 micrometer (at 30 keV). Depending upon the particular application and specimen composition, the operator optimizes the proper conditions for magnification range, by adjustment of accelerating voltage, beam current and spot diameter.

The electron beam is precisely deflected in the raster pattern either in an analog or digital manner depending upon the design of the particular instrument. Most newer instruments employ digital scanning so that they can use frame storage and also incorporate auto-focus and auto-astigmatism correction [10,11]. This deflection is synchronized with the deflection of the display cathode ray tube (CRT) so there is a point by point visual representation of the specimen on the CRT screen as the electron beam scans the specimen. The smaller the area scanned by the electron beam, in the raster pattern relative to the display CRT size, the higher the magnification. The theory of the operation of the scanning electron microscope has been covered by several authors [9,12–14] and the reader is directed there for more in-depth coverage of this topic.

## Electron Signals Used for Metrology

The primary electron beam, as it traverses the sample, interacts directly with the sample resulting in a variety of signals being generated that are useful for semiconductor inspection, analysis and metrology [15]. For historical reasons the major signals of interest to microelectronics dimensional metrology are divided into two groups, backscattered and secondary electrons, even though it must be remembered that this distinction is often arbitrary, especially at low beam energies.

### Backscattered Electrons

**Figure 4:**
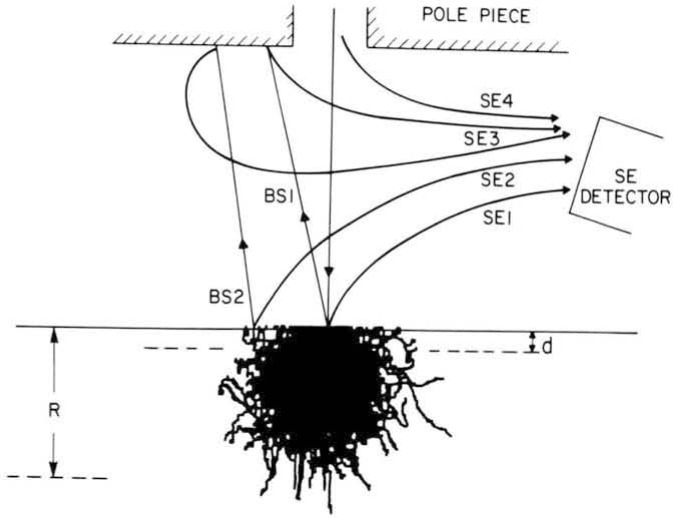
The origins of various components of the secondary (SE) and backscattered (BS) electrons in the specimen chamber of the SEM. The electron range in the specimen is R, and the secondary electron escape depth is shown as d.

Backscattered electrons are those which have scattered within the specimen and have been re-emitted from the specimen surface with energies which are a significant fraction (50% or more) of the incident beam energy. On a typical specimen, between 10% and 30% of the incident electrons ultimately become backscattered electrons. This fraction varies with the atomic number and surface geometry of the specimen but it is relatively independent of the beam energy. Because these electrons have relatively high energies they can travel significant distances through the sample and emerge from the whole area defined by the beam interaction volume. Thus, in silicon at 15 keV a backscattered electron may escape from an area which is about one micron in radius and from depths of up to one and a half microns beneath the surface (fig. 4). The maximum range of electrons in a sample, can be approximated using the expression derived by Kanaya and Okayama [16]

$$Range(\mu m)=0.0276AE_{0}^{1.67}/Z^{0.889}\rho$$ (2)

where *E*_0_ is the primary electron beam energy (keV), *A* is the atomic weight, *ρ* is the density of the material (g/cm^3^) and *Z* is the atomic number. The calculated range of electrons in silicon for a variety of changes in accelerating voltage is shown in table 5. If one considers that the calculated range approximates the boundaries of the electron trajectories as a region centered on the beam impact point (fig. 4), then it can be seen that the backscattered electrons which emerge from approximately the upper one-third to one-half of this region do not, in general, carry much information about the high resolution details making up the surface topography of the specimen. But, at low magnifications (less than 1000×) where features on the scale of microns are being viewed, significant and useful signal information is carried by these electrons.

Because the backscattered electrons are energetic they are re-emitted away from the sample surface in straight lines. Consequently, they are usually collected by placing a detector in their path rather than by using a collecting (attracting) field. The size, sensitivity and position of the detector drastically affect its collection efficiency and thus the appearance of the image and, of course, the results of any measurements made from it. A large detector placed above the sample will give a high quality, low noise, image that appears evenly illuminated but in which the topography is of low contrast. A small detector, placed to one side of the sample, will collect fewer electrons (yielding a noisier image), but will produce topographic contrast that is much stronger and is marked by what appear to be strongly directional shadows. Metrology schemes must, therefore, take into account the characteristics of the detector and its effect on the observed signal.

### Secondary Electrons

**Figure 5:**
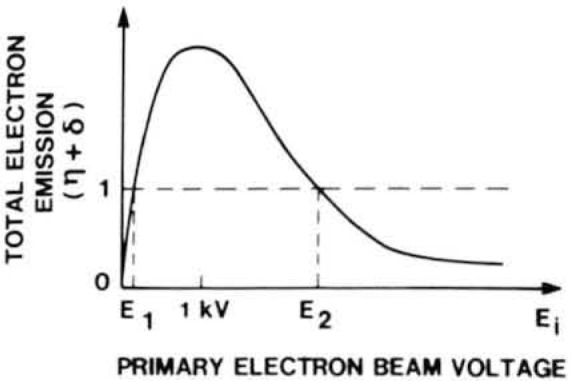
Variation of total secondary plus backscatter electron yield from a specimen plotted as a function of incident beam energy. The total yield is unity for two energies E-1 and E-2 called the cross-over points.

Secondary electrons are another signal of interest in the SEM. These electrons are defined as those with energies between about 1 and 50 eV. At an incident energy of 15 keV each 100 incident electrons will produce, on average, 10 to 20 secondary electrons. This number, however, increases rapidly as the beam energy is reduced until at some energy E-2 (fig. 5) the total secondary plus backscattered yield (*n* + δ) becomes one (unity); that is to say each incident electron produces on average one emitted electron. Since the secondaries are low in energy, their trajectories are readily deflected by local electric or magnetic fields. High efficiency collection of secondaries is therefore possible even with a physically small detector since this can be made efficient by applying a suitable electron-attracting (biasing) voltage to it. This convenience plus the higher signal-to-noise ratio has led to secondary electrons being the preferred mode of operation for most purposes in the SEM.

Because of their low energy, secondaries cannot reach the surface from deep in the specimen, and typically they escape from a region only 5 to 10 nanometers beneath the surface. They, therefore, carry surface-specific information. Several different types of secondary electrons can be distinguished [17], as shown in figure 4. The most desirable for metrology and imaging are called the SE1 electrons, which are generated as the beam enters the sample. These secondary electrons are produced at the beam impact point and therefore carry the highest resolution information. The secondary electrons that are produced by backscattered electrons as they again pass through the surface escape region are called SE2 electrons. These secondaries are emitted from a surface area as large as that from which the backscattered electrons emerge, and the number of these electrons will depend directly on the number of backscattered electrons. Thus, the SE2 signal carries the same contrast information, and displays the same spatial resolution, as the backscattered signal. Typically, the SE2 component is as large, or larger than, the SE1 signal.

Finally, secondary electrons can also be produced external to the specimen by backscattered electrons which have been emitted from the specimen that hit the polepiece or walls of the specimen chamber (SE3), or from the impact of the incident electrons on the electron-optical defining apertures (SE4). The SE3 electrons carry information similar to that of the SE2 electron signal. The SE4 electrons contribute no contrast information, but, simply act as a “background” to the wanted signal, reducing its visibility and signal-to-noise ratio. Thus, in an SEM designed for metrology, attention must be given to reducing the relative magnitudes of the SE3 and SE4 components. In an unoptimized instrument, as much as 60% of the total secondary signal collected can be attributed to these unwanted emissions.

Since the secondary electron signal is easily influenced by the application of local electrical or magnetic fields, it is readily understood that the collection efficiency of a detector can relate directly to its position and potential. Detectors that have a location at some off-axis angle, as in many instruments also equipped to do x-ray microanalysis, show preferentiality of detection. In these cases, it is not possible to achieve the symmetrical waveforms necessary for precise linewidth metrology. To compensate for an off-axis position of the secondary electron detector, on a sample normal to the electron beam, the sample must be physically rotated toward the detector until the video waveform of the line becomes symmetrical, then the structure can be straightened on the display CRT by adjusting the raster pattern with digital raster rotation. Since error can be introduced using this technique during the measurement of a tilted sample, it is much more desirable to have an on-axis detector [6] or two similar detectors on either side of the sample and the signals balanced and summed [18].

## Low Accelerating Voltage SEM Operation

Historically, scanning electron microscopy was done at relatively high accelerating voltages (typically 20–30 keV) in order to obtain the best signal-to-noise ratio and best resolution. Nonconducting or semiconducting samples required an overcoating of gold or a similar material to provide conduction to ground of the electrons and to improve the secondary electron generation of the sample. In semiconductor device processing, this procedure is considered a destructive technique because the device cannot be processed further. On-line inspection during the production process of semiconductor devices is designed to be nondestructive which requires that the specimen be viewed in the scanning electron microscope uncoated. A thin insulating film on a conducting substrate can be viewed at a high accelerating voltage with an absence of electrical charging since most of the electrons are deposited in the substrate, but not all films are sufficiently thin for this technique. High accelerating voltages can also damage a semiconductor sample or device [19]. Low accelerating voltage inspection is thought to eliminate, or at least minimize, charging and device damage. In order to accomplish this in the SEM, the sample is viewed at accelerating voltages in the range of about 0.2–2.5 keV. Further advantages derived by operating the SEM at low accelerating voltages are that the electrons impinging on the surface of the sample have less energy, penetrate into the sample a shorter distance and have a higher cross section for the production of secondary electrons near the surface where they can more readily escape and, thus, be collected.

The secondary electrons are the most commonly detected signal carrier for low accelerating voltage inspection since their signal is much stronger than any of the others. The behavior of the total emitted electrons from a sample, shown in figure 5, is extremely significant to low accelerating voltage operation as those points where the curve crosses unity (i.e., E-1 and E-2) are the points where no electrical charging of the sample will occur. During irradiation of an insulating sample such as photoresist or silicon dioxide viewed normal to the electron beam, a negative charge can develop causing a reduction in the primary electron beam energy incident on the sample. If the primary electron beam energy is 10 keV and the particular sample has an E-2 of 2.0 kV then the sample will charge to about −8 kV so as to reduce the effective incident energy to 2 keV and bring the yield to unity. This charging phenomenon will have detrimental effects on the electron beam and degrade the observed image (to be discussed later). If the primary electron beam energy is chosen between E-1 and E-2 then there will be more electrons emitted than are incident in the primary beam, and the sample will charge positively. Positive charging is not detrimental as it is only limited to a few electron volts because of the resulting barrier to the continued emission of the low energy secondary electrons. This reduction in the escape of the secondaries stabilizes the surface potential but reduces the signal as these electrons are now lost to the detector. The closer that the accelerating voltage approaches to the unity yield point, the less the charging effects. Each material component of a specimen being observed has its own total emitted electron/keV curve and so it is possible that in order to completely eliminate sample charging a compromise must be made to accommodate the different specimen materials. For most materials used in present semiconductor processing an accelerating voltage in the range of about 1.0 keV (±0.5 keV) is sufficient to reduce charging and minimize device damage. Tilting the sample increases the total electron emission and thus, is also useful in decreasing sample charging (to be discussed later).

Although operation at low beam energies is useful for the inspection of delicate samples with a minimum of charging, the filament brightness is lower leading to reduced signal-to-noise ratio. This results in a loss in apparent sample detail. High brightness electron sources and digital frame storage techniques for signal integration over short periods of time at TV rates minimize this problem [20]. The more abiding problem with low accelerating voltage operation is the lower spatial resolution (as compared to the higher beam energy operation) characteristic of this operational mode. If a contemporary instrument, equipped with a high brightness lanthanum hexaboride filament is capable of 4 nanometers resolution at 30 keV accelerating voltage it may be only able to achieve about 10–12.5 nanometer resolution at 1.0 keV. This limitation must be understood and factored into the precision requirements for submicrometer measurement applications.

## Specimen Beam Interactions

**Figure 6:**
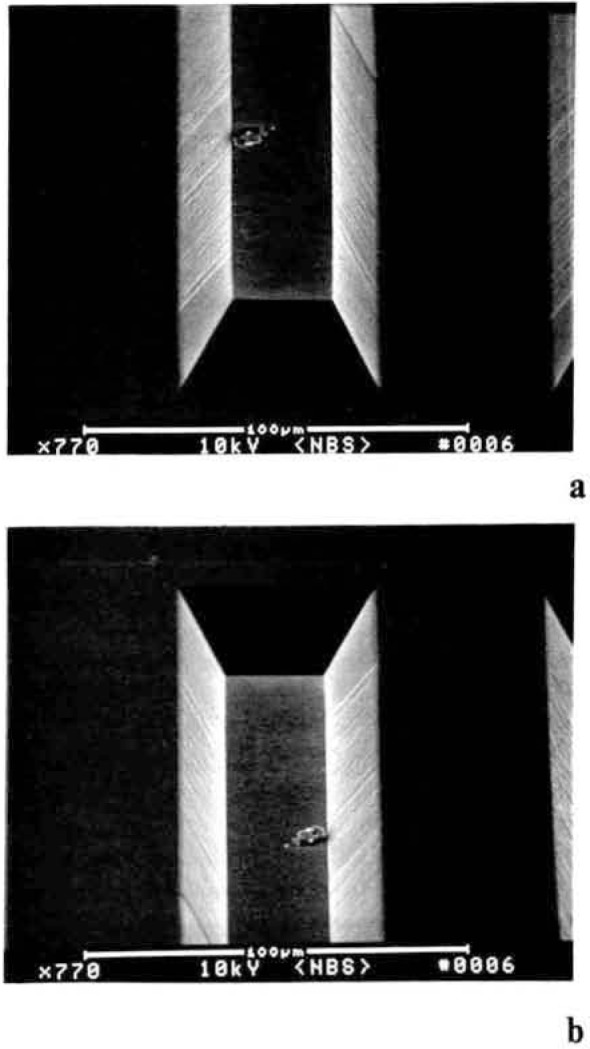
Scanning electron micrographs showing an illusion possible in the SEM that demonstrates that an understanding of the sample is often necessary to facilitate proper interpretation of the images. (a) In this micrograph, the image appears to be a line standing above the substrate. (b) In this micrograph, the structure appears as a trench. The only difference between these micrographs is 180 degrees of raster rotation.

While it is often true that the appearance of a scanning electron micrograph is such that its interpretation seems simple, this may not always be the case (figs. 6a and 6b). Care must always be taken so as not to become confused by “obvious” interpretations. When quantitative feature-size measurements are to be made it is even more necessary to be able to unambiguously relate signal variations to the details of the surface morphology. Because the interaction of electrons with a solid is such a complex affair (e.g., each electron may scatter several thousand times before escaping or losing its energy, and a billion or more electrons per second may hit the sample) statistical techniques are an appropriate means for attempting to mathematically model this situation. Although transport theory [21] provides an elegant solution for simple systems, it is of little value when considering complex device geometries. The most adaptable tool, at the present time, is the “Monte Carlo” simulation technique. In this technique, the interactions are modeled and the trajectories of individual electrons are tracked through the solid. Because many different scattering events may occur, and because there is no a priori reason to choose one over another, algorithms involving random numbers are used to select the sequence of interactions followed by any electron (hence the name, Monte Carlo). By repeating this process for a sufficiently large number of incident electrons (usually 5000 or more) the effect of the interactions is averaged, thus giving a useful idea of the way in which electrons will behave in the solid.

The Monte Carlo technique has many benefits as well as several limitations [6,22]. Because each electron is individually followed, everything about it (its position, energy, direction of travel, etc.) is known at all times. Therefore, it is straightforward to take into account the sample geometry, the position and size of detectors, and other relevant experimental parameters. The computer required for these Monte Carlo simulations is modest and, in fact, even current high performance personal computers can produce useful data in reasonable times.

In its simplest form [23,24], the Monte Carlo simulation allows the backscattered signal to be computed, since this only requires the program to count what fraction of the incident electrons subsequently re-emerge from the sample for any given position of the incident beam. By further subdividing these backscattered electrons on the basis of their energy and direction of travel as they leave the sample, the effect of the detection geometry and detector efficiency on the signal profile can also be studied. However, while this information is a valuable first step, under most practical conditions it is the secondary electron signal that is most often used for metrology in the low accelerating voltage applications. Simulating this is a more difficult problem because two sets of electron trajectories— 1) those of the primary (incident) electron, and 2) those of the secondary electron that it generates—must be computed and followed. While this is possible in the simplest cases [7,25] it is a more difficult and time consuming approach when complex geometries are involved.

For this reason, a new approach has been proposed [8,22] and is currently undergoing further development. In this method, a simple diffusion transport model for the secondary electrons is combined with a Monte Carlo simulation for the incident electrons. This procedure allows both the secondary (SE1+SE2) and the backscattered signal profiles to be modeled simultaneously with very little increase in computing time. Once that data are available, the effect of other signal components, such as the SE3 signal, can also be estimated. All the computed results discussed below are generated using this method.

The importance of being able to model signal profiles for some given sample geometry is that it provides a quantitative way of examining the effect of various experimental variables (such as beam energy, probe diameter, choice of signal used, etc.) on the profile produced, and gives a way of assessing how to deal with these profiles and determine a criterion of line edge detection for given edge geometries and thus, a linewidth [6]. However, at the present time, the Monte Carlo technique is is not useful for deducing the line-edge geometry from the acquired SEM video profiles.

## SEM-Based Metrology

The basic premise underlying the use of the scanning electron microscope for critical dimension measurement for semiconductor research and production applications is that the video image acquired, displayed, and ultimately measured reflects accurately the structure of interest. However, the secondary electrons detected do not necessarily originate at the point of impact of the primary electron beam. Indeed the effects of the four types of electron contributions to the actual image or linewidth measurement (see fig. 4) have not been fully evaluated. Errors in measurement are also introduced by sample charging and environmental influences (e.g., stray magnetic fields and vibration). In measurement applications, error due to the actual location of signal origination usually will not affect pitch measurements because the errors cancel [1,26,27]. However, in linewidth measurement, many potential errors are additive and thus will give twice the edge detection error to the measured width. The imprecision of any SEM-based metrology system is composed of two basic components: the imprecision of the actual instrument itself assuming an ideal sample, and the imprecision introduced by variations in the actual sample [28]. Some of the factors that today limit the precision of the SEM metrology instrument will now be discussed.

## Definition of Linewidth

**Figure 7:**
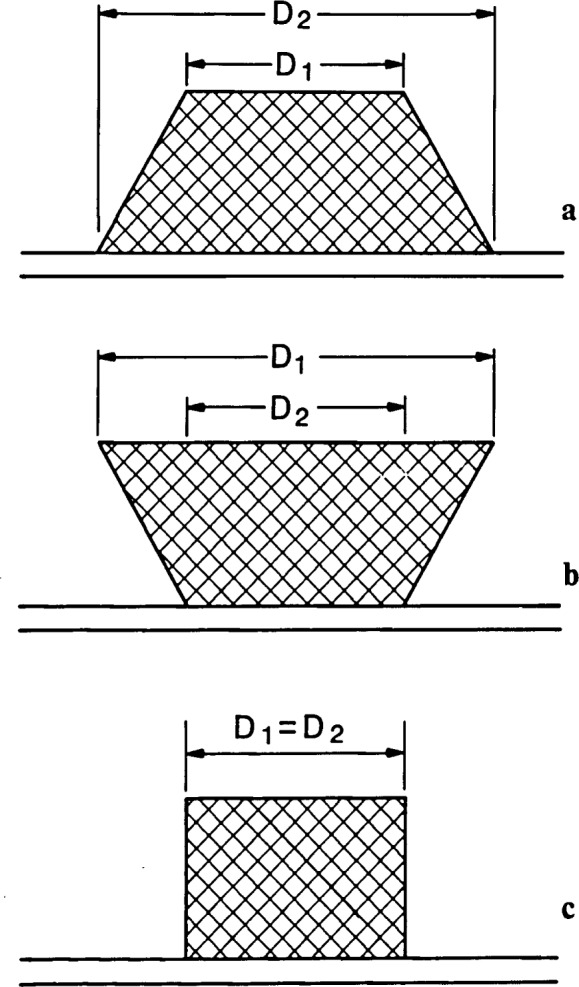
Drawing of a line structure as viewed in cross section showing the confusion possible in determining what edge is, in fact, being measured in the scanning electron microscope. (a) Trapezoidal structure where the upper width D1 is smaller than the base width D2. (b) Undercut structure where D1 is larger than D2. (c) Structure with vertical sidewalls where D1 and D2 are approximately equal.

Scanning electron microscope metrology and optical metrology have one thing in common at the present time; that is except for vertical edges, there is no well-defined definition of the meaning of linewidth [1]. The first consideration that must be developed and defined when describing the term linewidth is what is actually being physically measured. Depending upon the lithographic process, the definition of linewidth may vary relative to the structural importance to subsequent steps. Figure 7a shows an idealized structure in cross section. In this case, D1 and D2 are not equal and hence the sidewall has some angle from normal. Linewidth could be defined as D1 or D2 or their average. Due to the large depth of field of the SEM inspection instrument, this distinction becomes significant since, if the conditions are properly chosen, both regions could be simultaneously in acceptable focus. Another situation for linewidth definition error occurs when an undercut sample is being observed (fig. 7b). In this case, D1 is smaller than D2, but D1 may not be readily observed unless the sample is highly tilted. Either of these two cases can result in difficulties in deducing where the edge is located and errors in precision. As the sidewall approaches 90 degrees (fig. 7c) this definition problem diminishes as D1 = D2 and precision (reproducibility) problems relate only to edge and sidewall irregularities and not misinterpreted edge location. A further confusion to any of the above instances would be introduced if the line was asymmetrical in cross section. In addition, the improved resolution of the SEM, as compared to the optical microscope, can also lead to deceptively imprecise data due to small irregularities in edge and sidewall structure that can be resolved and measured by the SEM. This discussion of the definition of linewidth has been limited to the description of where on the particular structure the measurement is to be made and not how to make the measurement. Further work modeling the structures and relating it to the physical edge is necessary before the actual linewidth can be defined and accurately measured.

## Sources of Instrumental Error

### Methods of Measurement

In commercial SEMs, used for critical dimension (CD) or linewidth metrology, two basic techniques of measurement are presently employed: beam scanning and frame storage. The two techniques are, in principle, similar. The beam scanning technique digitally acquires one scan line of video information from a sample positioned perpendicular to the x direction (horizontal scanning axis; the y-scan direction is commonly the vertical axis) with some pixel point resolution, and measurement algorithms are arbitrarily applied to that single line scan to obtain the width. Multiple acquisition of these linescans enables averaging over the field of view. In the frame storage imaging and measurement technique, an entire raster of information is stored digitally at some pixel point resolution depending upon the hardware design of the particular instrument. With this technique, since many individual line scans of data are actually stored (generally in about 512 positions along the line in the y direction) measurement algorithms can be applied anywhere in the field to data acquired in the x direction. Under both of these conditions, the precision of the measurement is severely influenced by the factors previously discussed such as electron beam effects, sample irregularities and the definition of linewidth. The instrumentation design and limitations must also be considered as a factor adding uncertainty to the measurement. For example, scan linearity, magnification compensation, and lens hysteresis are serious influences that must be considered, understood and compensated for, if possible, to name a few. Jensen 1980, Jensen and Swyt 1980, Seiler and Sulway 1984 and Nyyssonen and Postek 1985, discuss these and other instrumental limitations (e.g., CRT linearity) and the reader is directed to these references for further information. The overall precision of the metrology system is also limited by the pixel point resolution of the measurement system. Table 6 demonstrates the linewidth measurement uncertainties associated with a 512×512 pixel point resolution system. Many commercial linewidth measurement systems at the present time acquire approximately 512 pixel points of information for linewidth measurement although some of the newer “dedicated” systems can acquire up to 2048 pixel points of information [4]. These techniques, even with their limitations, are of value due to their speed as throughput is a major concern for the production engineer. However, limitations on the pixel point resolution must also be understood in order to properly interpet the measurement results.

Measurements can also be done by moving the stage/sample rather than the electron beam [6,26]. In this technique, the beam remains stationary (or oscillated slightly in the y direction to integrate slight sample irregularities) and the sample is driven in the x direction on a piezo stage. As the sample is moved, its position is precisely monitored using laser interferometry. Both the sample position and video intensity data for each point are stored for analysis. Using this technique, most of the errors in the SEM focusing and scanning system are minimized if not eliminated (but not the electron beam/sample interaction problems) and the measurement can be referenced to an accepted standard of length traceable to national standards [29]. Unfortunately, this technique although extremely accurate requires an elaborate laserinterferometer piezo-scanned specimen stage. Consequently, the procedure is relatively slow, thus making it unattractive for most production situations where throughput is of paramount importance.

### Environmental Influences

The scanning electron microscope metrology system used for on-line inspection is usually located in a clean room. A great mass of literature is available on the air scrubbing aspects of the clean room and the mechanisms necessary to ensure that particle counts are low. However, little attention has been paid to the consequences of these actions on the metrology instrumentation. The SEM metrology instrument is an imaging system and as such the problems posed by the clean room environment are readily observable by these systems with excellent resolution. It should be noted that these problems can also detrimentally affect *other* clean room instrumentation but their effects are not directly observable in time and so the significance is lost. In most cases surveyed, the SEM metrology instruments presently operating in the typical clean room are not performing optimally. This is usually due to two main reasons: excessive vibration and stray electromagnetic fields.

### Vibration

**Figure 8:**
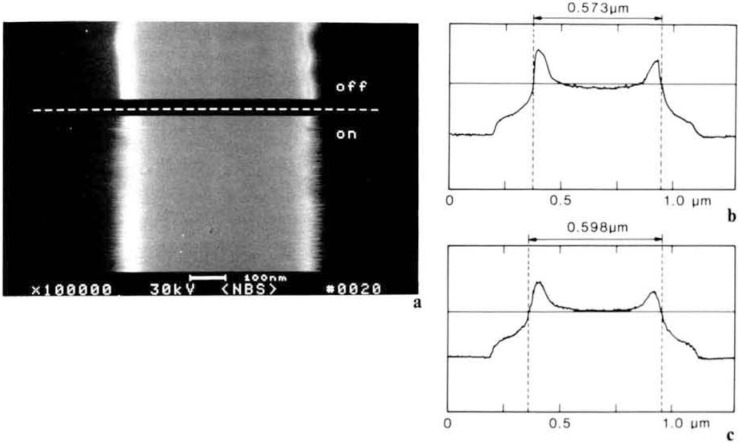
The effect of deliberately induced vibration on the image and measured linewidth. (a) Scanning electron micrograph showing the effect of vibration induced by a small cooling fan on the image; source off (top) and on (bottom) on the image. (b) Typical linewidth measurement taken with an arbitrary 40% positive automatic threshold crossing algorithm under ambient vibration levels typical for proper SEM operation. (c) Similar measurement, using the same threshold crossing algorithm, of the same sample position after vibration was induced. (100,000×; 30 keV)

The effect of vibration on linewidth metrology, while obvious, is unfortunately, often overlooked. Clearly, vibration can originate from either the instrument or the environment, but their effects on the measurement of linewidth are similar (figs. 8a and 8b). Vibration, of the specimen relative to the electron beam, broadens the measurement and yields a linewidth uncertainity of twice that of each edge. The sources of vibration in the particular installation must be identified and eliminated or steps taken to isolate the instrument from them. Some of the typical sources of vibration in the clean room are: undampened floor vibration, blower fans, vacuum pumps and air flow across the instrument. One solution to the vibration problem is to decouple the clean room from the measurement instrument either by placing the instrument on a vibration isolation unit, or a massive concrete pillar sunk to bedrock, or both. Of the two possibilities the latter is preferred wherever possible. The concrete instrument pad can then be properly vibration isolated from the clean room floor. It is recommended, that the entire instrument including the area used by the operator be on the concrete pad and not just the column section as vibrations can be transferred via the operator and umbilicals to the column section. Unfortunately, there is some cost to this modification but at some point decisions to optimize the metrology instrumentation must be made to ensure that the required measurement precision be met. Vibration induced by air flow can be minimized or eliminated by instrument shrouding or shielding. One consequence of unrecognized vibration is deceptively good measurement system precision since the continuous vibration is being continually integrated into the image, obscuring the actual sample detail and, smoothing the measurement data. Probably the best solution to the metrology problems is to design clean rooms that have the SEM metrology instrumentation in an optimized external, but adjacent, area to the actual clean area and the product transferred to it in a controlled manner.

### Stray Magnetic Fields

**Figure 9:**
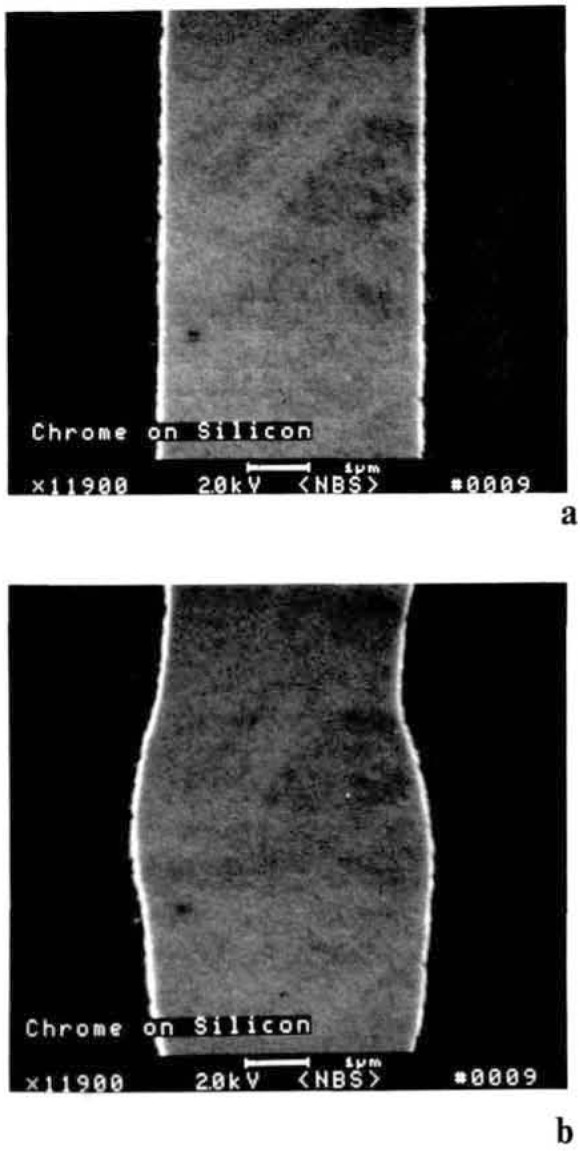
The effects of non-synchronized ac field induction on the image in an SEM. (a) Micrograph taken under ambient conditions where the instrument is synchronized to line (60 Hertz). (b) Micrograph taken after induction of a non-synchronized field that is 1/4 cycle off of 60 Hertz. Note the broadening of the structure.

The SEM metrology instrument is an electronic instrument in an electronically hostile environment [30]. Both ac and some dc fields can affect this instrument in an undesirable manner. Studies have shown that many problems involve a field effect induced by the improper wiring and grounding of the instrumentation [31,32]. This not only refers to the SEM but also to any other instrument or wiring in the immediate environment including the lighting system Ground looping in the clean room of equipment and lighting can result in ac fields in excess of 30 milligauss at the SEM column This is equivalent to operating a small transformer a few centimeters from an SEM column Many SEM manufacturers specify that external fields not exceed 1–3 milligauss for proper instrument operation Newer instruments operating at TV rates synchronize the scan to the 60 Hertz (i.e.. power line) thus concealing the observable field effects of that component on the CRT screen. Further, not all of the interference is generated at that frequency and problems can still be induced in the image and the measurement (figs 9a and 9b). The most effective approach to the problem of undesirable stray fields is to identify the source and to eliminate it there Supplemental shielding should only be used afterwards if the sources cannot be identified or eliminated. The shield may only prove to be a temporary solution since the overall complexion of the situation may be altered as other equipment is moved in and out of the clean room environs over time.

### Operator Factors

Scanning electron microscopes, especially those equipped as metrology instruments, are complex, expensive investments. One area that has been severely neglected by many semiconductor companies is the role the metrology instrument operator plays in the success or failure of the on-line inspection program. Even the simplest of the SEM systems are far more technologically involved than their optical microscope counterparts (although in both instances highly trained individuals should be used). This is especially true where routine instrument maintenance is concerned. Not every applicant is suited to become an SEM metrologist, and once an appropriate candidate is selected, a substantial amount of training must be invested in order for that individual to become confident with the particular instrument or instruments under his supervision. Further, once an individual has proved to be an asset in that position he must be encouraged to remain in that area and not be transferred out. Once an operator leaves the SEM metrology area his real experience value is lost. Experience cannot be taught, only gained! The trend toward automation of the SEM inspection processes may minimize the need for a large number of trained operators at some point in the future; however, this will not be for some time.

### Instrument Maintenance

The SEM requires periodic electron optical column maintenance in order to maintain proper performance. Proper maintenance is especially important to low accelerating voltage operation. The maintenance period varies with instrument design, application and the types of specimens observed. It must be noted that in all instruments the components that directly interact with the electron beam (e.g., apertures) do become dirty due to deposition of residual hydrocarbons and oxidation products [33]. In a clean vacuum system, the majority of these contaminants are outgassing products of the sample. Contaminant build-up can result in charging in the electron gun or in the column resulting in poor performance [34]. Asymmetrically deposited contamination, especially on apertures, increases astigmatism levels and may ultimately lead to the point where it becomes uncorrectable. Also, heavy build-up of contamination on an aperture can dislodge and either block the beam path or develop a charge and deflect the beam. The instrument operator must be experienced enough to recognize this condition and suspend work and take corrective actions so as not to compromise the measurement work. Some maintenance downtime must be expected on a periodic, or on an as-needed, basis in all production situations. Instrument manufacturers consider routine maintenance to be a user responsibility; however, in recent years this has been relaxed somewhat due to extended service policies and improved instrument performance. In order to regain the original performance level, only trained, experienced personnel fully understanding the work should undertake routine maintenance. Otherwise, extended and costly downtime may result.

One problem associated with the SEM in the production environment has been the lack of unified instrument standardization techniques that ensure that an instrument is operating optimally or, once an instrument has been dismantled for routine maintenance, that it is brought back to the same optimum level of performance where it was once running. Further, the data taken during the interface time between routine maintenance periods or while a decision was being made to service an instrument may, or not may not, be characteristic of the actual product, but a reflection of the condition of the instrument. Clearly, critical decisions must be made by the operator, based on the experience with the particular instrumentation in place that affects product acceptance. This is especially troublesome in locations where multiple instruments are in place (especially if they are from several different manufacturers) and the data is fed into a central data base for real-time analysis. Techniques for this purpose must be developed and diagnostics must be implemented into the SEM metrology instrument for this purpose. Each day, or at the beginning of each shift, diagnostic procedures must be done to ensure that the instrument is performing properly.

### Sample Charging

**Figure 10:**
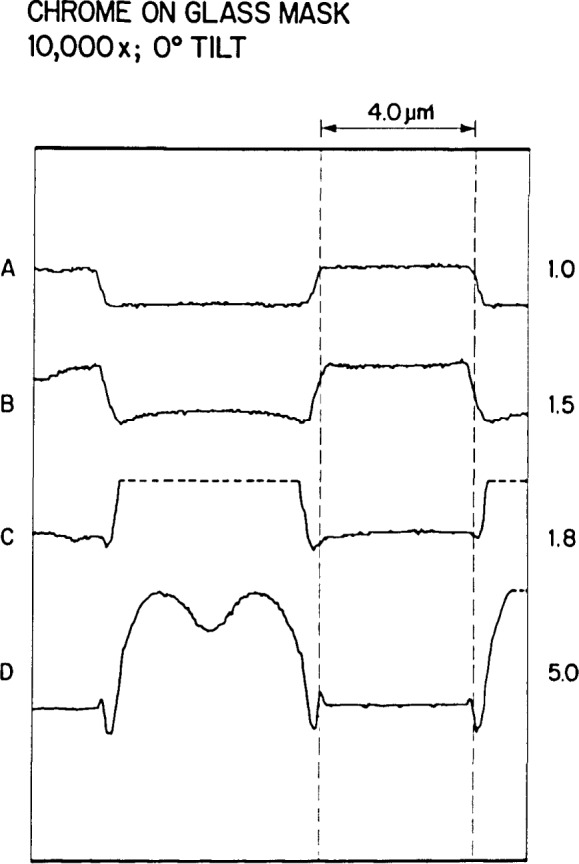
Sample charging and the effect on the video profile. This chrome-on-glass mask was viewed and measured at increasing accelerating voltages. At 1.0 keV (A) no apparent sample charging occurs, as the voltage was increased to 1.5 keV (B) charging in the glass area begins to occur. The increase of accelerating voltage through 1.8 keV (C) to 5.0 keV (D) results in apparent sample charging and over-ranging of the video signal (dotted line). Note how the profile in the measured area of the chrome also changes with accelerating voltage.

The effects of sample charging on measurements made in the SEM have been studied [35–37]. Negative charging resulting when the electron beam voltage exceeds E-2 (fig. 5) can affect the video profile (fig. 10) and thus the measurement. The foremost effect is the possible deflection of the electron beam as the sample builds up an appreciable charge with its accompanying electric field. This may either manifest itself as a catastrophic and obvious beam deflection where the image is lost or a more subtle and less obvious effect on the beam. The subtle effects are the most damaging to metrology as they may manifest themselves either as a beam deceleration or a small beam deflection. All instrument compensations directly relate to the accelerating voltage applied and all instrument adjustments (e.g., magnification) depend on this beam energy. A slight beam deflection around a line structure can move the beam a pixel point or two, thus invalidating the critical dimension measurement. One pixel point deflection of a 1 μm line measured at 10,000× with a 512 pixel point digital scan corresponds to about 38–40 nm linewidth error (less at higher magnification). Positive charging may also have detrimental effects on the measurements as a positively charging structure can attract secondary electrons from adjacent pixel points, thus altering the measurement waveforms.

Sample charging can be reduced, if not completely eliminated, by adjustment of the accelerating voltage to the appropriate points on the total electron emission curve (fig. 5). Rapid TV-rate or near-TV-rate scanning is also being employed by several manufacturers to further reduce charging. Under these conditions, the electron beam dwells on the sample for less time per point than in slow scan, thus the charge has less time to develop. Another possible charge reducing technique which offers some improvement, is to tilt the sample toward the detector. Tilting the sample permits operation at higher accelerating voltages without charging effects by increasing the total electrons emitted. A sample viewed at 45 degrees of tilt may not demonstrate charging with an accelerating voltage as high a 2.5 keV whereas the same sample will charge at about 1.3–1.4 keV viewed normal to the electron beam [37]. However care must be taken during the critical dimension measurements to minimize possible error that tilting may introduce [37].

### Signal Detection and Accelerating Voltage

**Figure 11:**
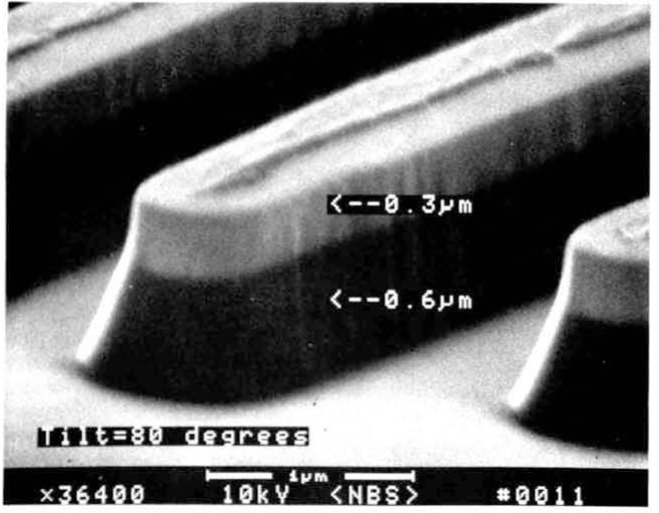
Micrograph of a nominal 0.75 μm line showing the silicide and the etched silicon layers.

**Figure 12:**
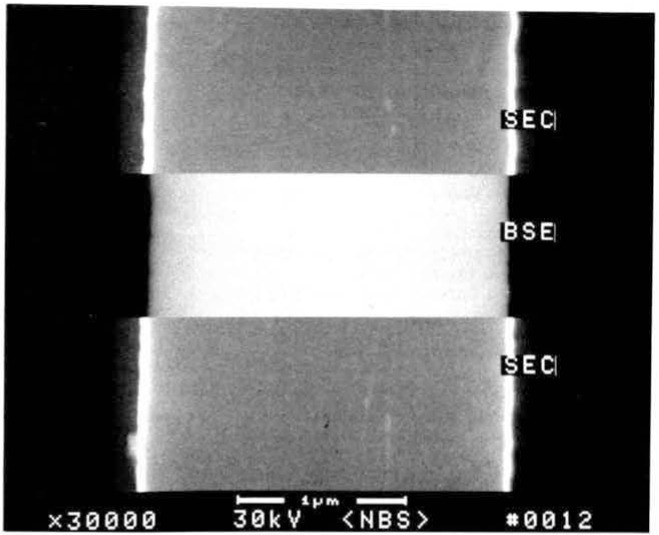
Effect of the mode of signal detection on the scanning electron microscope image. In this split field image, the effect of signal detection strategies on the image and thus the measurement, can be seen between secondary electron collection (SEC) and backscattered electron detection (BSE).

**Figure 13:**
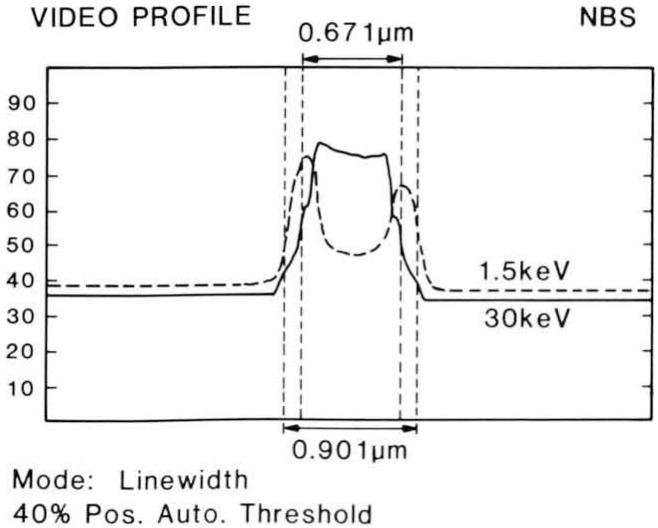
Overlay comparison of two digitally acquired video profiles of the 0.75 μm nominal line. One profile was taken at 1.5 keV and the other one taken at 30 keV. This comparison shows the reason for measurement discrepancies between accelerating voltages as the automatic threshold algorithm, arbitrarily set at 40%, is not appropriate for both the measurement conditions.

The magnitude of the errors introduced to the linewidth measurement relative to the mode of signal detection and of beam acceleration voltages has been studied [38]. Figure 11 shows a silicon wafer sample with a silicide layer patterned with micrometer and submicrometer lines. This sample was observed and measured under controlled conditions at a variety of accelerating voltages and electron detection modes. A micrograph showing the effect of the choice of signal detection (secondary and backscattered electron imaging) is demonstrated in figure 12. In that micrograph, the actual width of the line is not changing dimension as the beam scans it to the extent indicated, only the manner of perceiving it in the instrument changed. The results of repeated measurements with a pixel point resolution of approximately 9 nanometers demonstrate that, depending upon accelerating voltage applied and the electron detection mode used to image and measure the structure of interest, a variety of results can be obtained. Further, measurement broadening affects of the beam penetration and beam/specimen interactions are apparent. Figure 13 shows the video profiles of the line measured at two accelerating voltages and table 7 shows the measurement data. The SEM magnification was calibrated against an NBS standard and any processing irregularities present in the sample were well within the pixel resolution of the system and were also averaged over the field of view during the measurement process. Data was obtained from an average of 40 scans over a field of about 4.0 μm and the measurements between accelerating voltage changes were adjusted to give the pitch. This clearly demonstrates that measurement criteria for each accelerating voltage must be established so that electron beam effects can be properly accounted for. Changes in apparent dimension can be attributed to the uncertainties contributed by: electron beam interaction effects, solid angle of electron detection, detector sensitivity, and the criterion used to determine the edge location in the computation of linewidth. These data further suggest that if several instruments are operating on a production line, care must be exercised to insure that all are working with the same accelerating voltages, instrument and measurement conditions.

### Sample Contamination Effects

**Figure 14:**
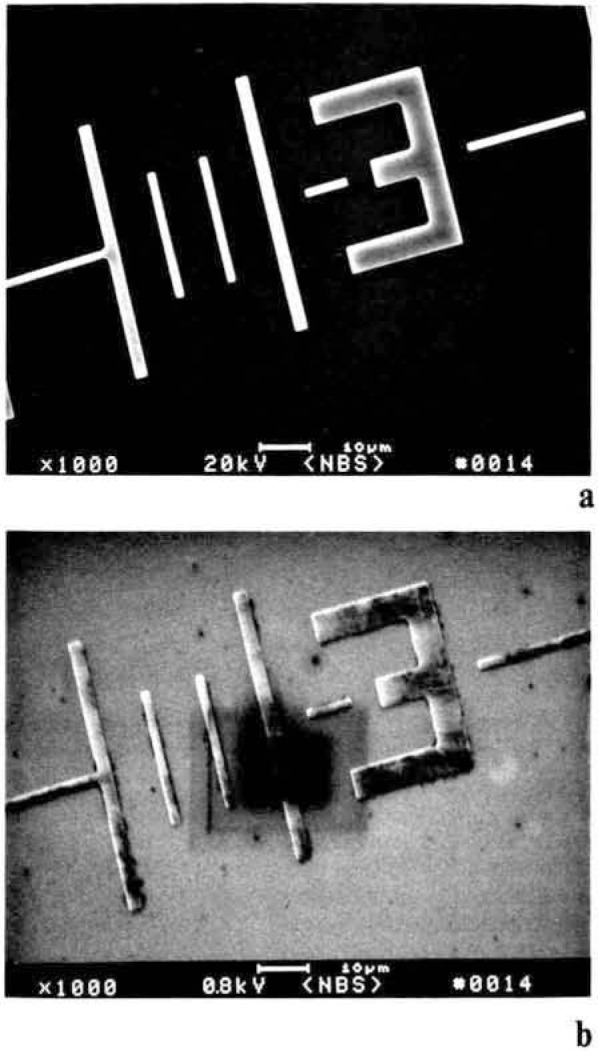
Sample contamination and the effects on the secondary electron image of a chrome on silicon wafer. (a) Sample viewed and photographed at high accelerating voltage (20 keV). (b) Sample viewed at low accelerating voltage (0.8 keV). Note the contamination on the ample apparent at low accelerating voltage operation is not apparent in the high accelerating voltage micrograph.

Semiconductor samples introduced into the SEM vary greatly in their surface cleanliness. For SEM inspection cleanliness, in this context, is not as much a lack of particles as a chemical cleanliness. This is as much of a concern in the SEM as it is in the optical microscope. The surface contamination levels present on the sample will vary with the preceding processing steps. Residual hydrocarbons adhering to the surface or diffusing from within the structures in the vacuum can ionize as the beam scans resulting in beam deflection or beam broadening. The electron beam can also act to decompose these hydrocarbons at the surface in the area of the raster pattern effectively depositing a layer of carbon (figs. 14a and 14b). At higher accelerating voltages the electron beam penetrates this contamination and shows little effect (fig. 14a) At low accelerating voltages used for non-destructive inspection this contamination can severely alter signal generation and thus compromise data.

### Sample Dimensional Changes

**Figure 15:**
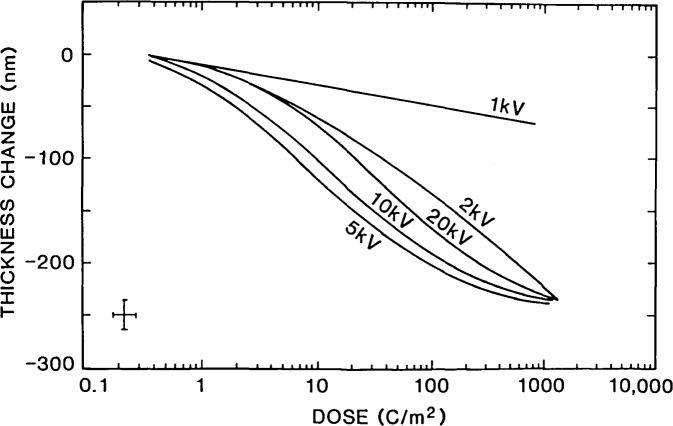
Experimental results showing the change in thickness of a 0.5 μm thick PMMA film under electron beam irradiation for several accelerating voltages. (Figure re-drawn from Erasmus [39].)

Electron beam irradiation can induce dimensional changes in photoresist structures [39–41]. A high resolution SEM image demonstrating a good signal-to-noise ratio can expose that sample to total electron beam dosages higher than that typical for electron beam lithography. This can have a pronounced affect on the critical dimensions by either causing the resist to swell or shrink. Erasmus (1986), recently studied the dimensional stability of several commonly employed resists. This work demonstrated that even with a beam operated at 1.0 keV accelerating voltage resist shrinkage can be induced. Figure 15 reproduces some of the results found for an easily damaged resist such as PMMA. The rate of resist shrinkage is greatest when the electron range is approximately equal to the thickness of the resist because, under irradiation, all of the beam energy is deposited in the resist. Clearly, this is an interesting and controversial topic and further work on this and other materials needs to be done. The possibility of dimensional changes of the sample occurring during the measurement process must be explored and care must be exercised to determine the optimum conditions where radiation damage and instrument operating conditions are optimized.

## Monte Carlo Modeling and Measurement

**Figure 16:**
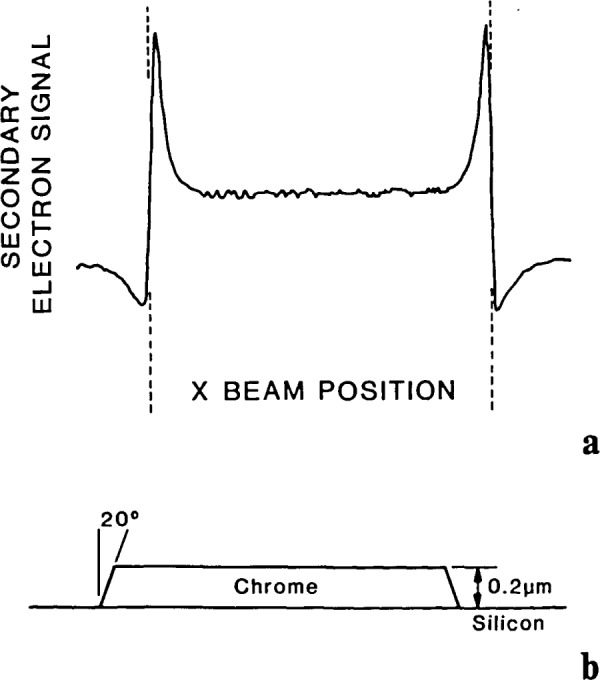
Monte Carlo modeling of SEM images. (a) Experimental video profile of the secondary electron image of the structure shown in (b) a 4.0 μm chrome strip on silicon. The incident beam energy was 10 keV.

The above discussion demonstrates that many factors contribute positively or negatively to scanning electron microscope metrology. Many of the previously identified influences can be modeled using the Monte Carlo technique in an effort to develop increased measurement accuracy and precision. The type of information to be gained from a Monte Carlo simulation of the linewidth profile is best illustrated using a real example. Figure 16a shows the experimental line profile obtained from a chromium strip, 4.0 μm wide and 0.2 μm thick, deposited on a silicon substrate (fig. 16b). The profile was recorded in the secondary electron detection mode at 10 keV beam energy, with the beam sampling the specimen at intervals of approximately 10 nanometers.

Using this geometrical information, and the relevant physical parameters (such as the density, atomic weight and atomic number) of the materials making up the structure, the expected signal profiles for the secondary and backscattered electron signals can be estimated using the Monte Carlo model. The profile is built up by performing the simulation for beams incident at points separated by 10 nm, in order to match the experimental pixel spacing. At each point 5000 trajectories are computed to ensure that the statistical error of the computation is kept to an acceptably low level. In order to generalize the simulation as much as possible, the profile is initially calculated for idealized conditions. Any given set of experimental conditions can then be matched by appropriate correction to this ideal profile.

**Figure 17:**
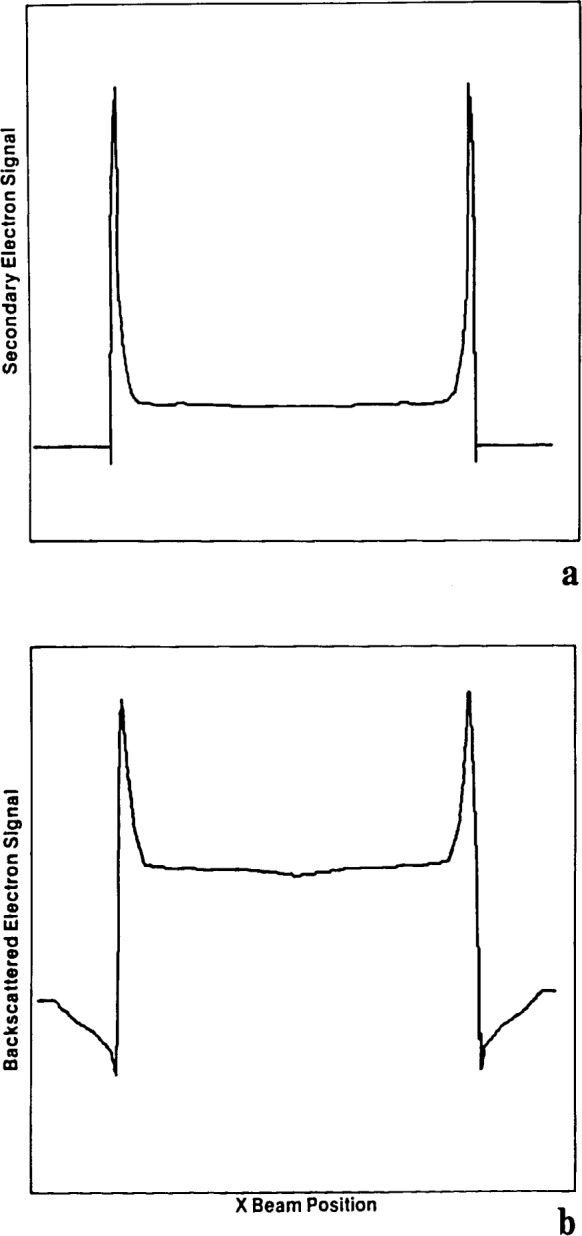
Monte Carlo modeling of SEM images. Idealized (a) secondary electron and (b) backscattered electron line profiles computed using the Monte Carlo technique for the structure shown in figure 16b.

The secondary and backscattered electron profiles obtained from the calculations are shown in figs. 17a and 17b. A comparison of these profiles with the experimental profile reveals several features of interest. The most important of these is the fact that the experimental profile, although recorded on the secondary electron mode, actually more closely resembles the computed backscattered profile. Compare, for example, the variation in signal just before the rapid rise at the edges of the chrome strip. The reason for this is that, as mentioned above, there are many sources of secondary electrons in the specimen chamber of the SEM. While, in principle, it is desirable to collect only those secondary electrons (SE1 and SE2) generated directly by the incident beam, in practice a contribution from the SE3 secondaries which are produced by the impact of backscattered electrons on the final lens and chamber walls are also included. These secondary electrons carry the information of the backscattered electrons that created them. The detected secondary electron signal is therefore actually a mixture of the secondary and backscattered components, the ratio of the mixture being determined by the exact geometrical arrangement of the sample in the chamber at any given time. For the data shown here, it is necessary to mix in about a 30% contribution from the backscattered electrons to match the experimental data.

Second, it is obvious that the features in the computed profiles are much sharper than those observed experimentally. One reason for this is that a real SEM has a finite probe diameter, while the computer model assumes a probe of zero size. The effect of a finite beam size can easily be simulated by convolving the computed profiles with a function such a gaussian, representing the size and intensity distribution of the incident electron probe. The computation also takes no account of the statistics of the signals detected. Because the measurement must be made in finite time, with a restricted beam current, the experimental data are shot-noise limited to a relatively poor signal-to-noise ratio. This can be modeled in the computed profiles by adding in an appropriate level of random noise. Finally, the computed profiles take no account of the properties of the electron detectors or the associated electronics. The effect of the behavior of these components can be mathematically modeled and then used to modify the simulated profiles.

**Figure 18:**
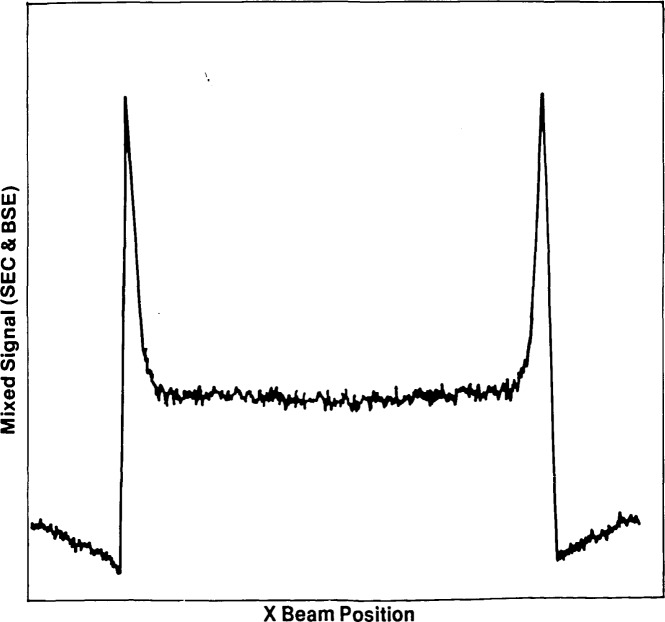
Stimulated line profile from the structure of figure 16b including corrections for finite beam diameter, detector efficiency, and signal-to-noise ratio. Components of both SEC and BSE signals have been added to allow for SE·3 contributions in the experimental profile (fig. 16a).

The final result of these modifications is shown in figure 18. The mixed secondary and backscattered signals have been convolved to an effective probe diameter of 25 nm full width half maximum (FWHM), adjusted to a signal-to-noise ratio of 10:1, and the detector efficiencies matched to those of the microscope. The resultant profile is now in good agreement with the experimental data. The advantage of proceeding in this systematic way from the idealized data to the fully corrected data is that it is possible to investigate the importance of different aspects of the experimental arrangement, by examining their effect on the linewidth “measured” from the computed profiles. For example, using an arbitrary 40% threshold crossing measuring criterion, the uncorrected secondary and backscattered profiles of figure 17 give widths that are, respectively, 0.45% and 0.95% smaller than the nominal expected width. After allowing for such factors as the finite probe size, the signal-to-noise ratio, and the detectors, the secondary profile now measures a value 0.5% larger than the nominal width, while the backscattered profile corresponds to a width 0.65% smaller. This significant discrepancy arises because the secondary and backscattered profiles are affected in opposite ways by the corrections applied. Although, for a line several micrometers in width the percentage error is not large, for a narrow line the effect would be proportionally much greater. Another result of this difference in width between the two profiles is that in situations where the experimental signal is actually a mixture of secondary and backscattered components, as in the case here, the measured linewidth will be a function of the ratio between the signals, and this may vary across the sample.

The sample discussed here is, in many ways, suited for SEM metrology since the feature is relatively large, has sharp edges, and is of high contrast. The fact that even in this case many sources of error are present indicates that the problems of more complicated specimens will be more challenging, and the requirement for modeling even greater.

## Automated Wafer Inspection

It is apparent the SEM metrology instruments will follow the direction of the present optical instruments and fully automatic inspection systems will become available. It would seem that all the components for such a system are presently available: electron beam column and components from SEM manufacturers, and high speed wafer and data handling systems from the optical instrument manufacturers. A joining of the two in inevitable. One must not be lulled into thinking that the two system strategies are directly interchangeable. There are serious differences in the physics of the two types of instruments that must be understood and dealt with before image analysis can acquire and decipher meaningful metrology data from the acquired electron image. From what has been shown in earlier parts of this paper the problems are not trivial.

A desirable feature in a fully automated wafer inspection/SEM metrology instrument is the ability to compare the acquired image to some stored image or image-generating data base and undertake linewidth measurement and analysis. It would be folly to think that an image acquired in an SEM could be directly compared to a CAD database until the electron beam/sample effects were fully understood. An image overlay based on the stored image of a good device at high pixel point density with that of the unknown could be implemented, however extremely tight controls on the instrumental data acquisition conditions (as discussed above) must be maintained otherwise false image differences would result.

The automated inspection tool, while computing linewidth, could also undertake particle and defect analysis. The SEM images with electrons. The ability to see a feature is a function of the contrast produced. If the contrast of the structure is not adequate it is not observed. Signal is directly related to the number of electrons provided by the electron gun and, in this instance, the image contrast is derived from at least two main sources: atomic number contrast and topographic contrast. The electron beam must supply sufficient electrons in a small enough gaussian spot to resolve the structure of interest and the particle must be observed at sufficient magnification so that it is clearly discernable from the background. Further, the measurement must be made at a magnification adequate to resolve the structural detail necessary to meet the precision specifications desired in table 1. For the modern IC processing applications, particulate matter with sizes down to the submicrometer region must be considered. Table 8 demonstrates a projected throughput vs. magnification for the analysis of a submicrometer particle for a typical chip size of 1 cm^2^. This analysis also assumes that there is sufficient atomic number contrast to image the particle, a pixel point resolution adequate to resolve it to the analysis system and sufficient beam current focused into a spot size less than a pixel point. It is clear that new data acquisition and data handling techniques necessary for this work will need to be developed in order that the SEM instrumentation compete with the throughput of present optical inspection instruments.

## SEM Measurement Standards

**Figure 19:**
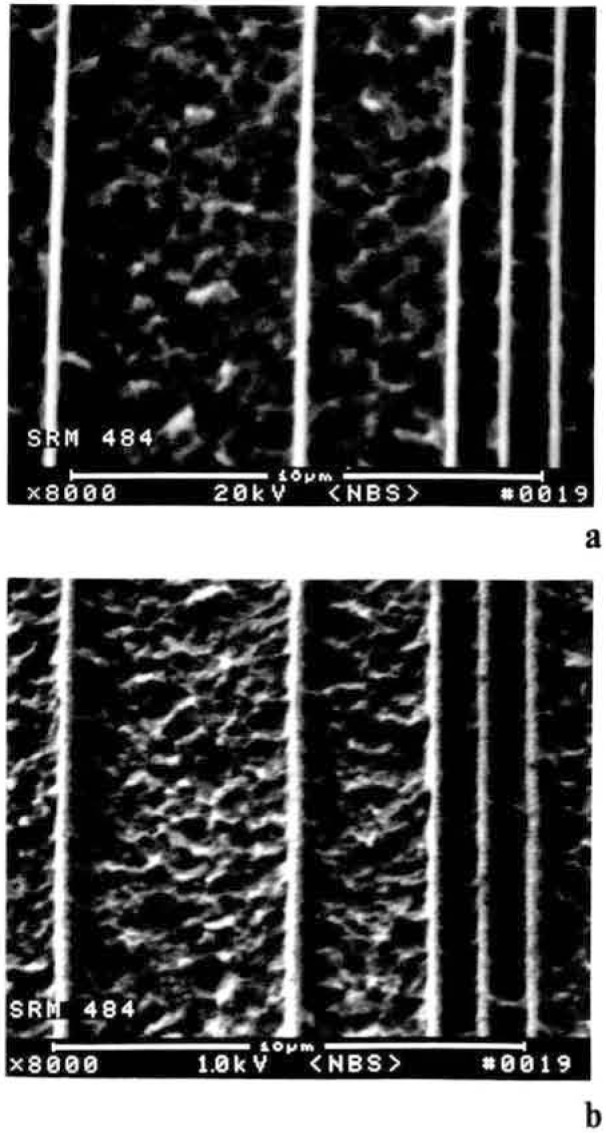
Scanning electron micrograph of the SEM magnification standard SRM 484 following the procedure used to enhance the contrast of the sample for low accelerating voltage use. (a) 20 keV accelerating voltage (b) 1.0 keV accelerating voltage.

A major project being undertaken at the National Bureau of Standards at the present time is the development of national standards for SEM linewidth metrology. The only magnification standard reference material (SRM) presently available for calibrating scanning electron microscopes is, SRM 484. This standard has served well for several years and is still useful for many SEM applications, but it was developed prior to the recent interest in low accelerating voltage operation and wafer inspection SRM 484, in its present form, is unsuitable for use in new SEM inspection instruments for two main reasons: a lack of suitable contrast in the 1.0 keV accelerating voltage range and the overall size which is not compatible with newly introduced wafer inspection instrumentation. Presently, a project has been initiated at NBS to physically modify this sample without altering its calibration or certification procedures to make it suitable for low accelerating voltage operation (figs. 19a and 19b). The linewidth measurement standard developed for the optical microscope SRM 474 is not designed or recommended for use in the SEM and it should not be used for this purpose [42].

The optical theory and modeling for the SRM 474 is not directly adaptable to the SEM and therefore the criteria used to determine the edge location is not applicable and should not be considered as such. From the above discussions of the electron beam effects and the requirement for modeling, this should be apparent as the two types of instruments are totally independent of each other in both the underlying physics and in operation. SRM 474 could, however, be used to measure pitch at low accelerating voltage under conditions where the sample is not charging. However, such use may damage the SRM (e.g., contamination) and render, it useless for optical microscopy. In this mode, the magnification of the instrument could be calibrated to pitch. However, again the reader is warned that continuing this adjustment process to include linewidth measurements is not recommended, as a general calibration procedure, because the edge criterion so obtained would only be valid for a similar chrome-on-glass mask.

For the present time, product precision is a prime concern to the semiconductor industry, and until such national standards for SEM linewidth measurement on integrated circuit wafers are available, the best that can be done now is the development of a series of internal “golden” samples within a particular organization for each level of processing [43]. The development of such samples referenced between the SEM and the optical microscope has been discussed [1]. Using the established national standards to properly adjust the magnification of an instrument, this series of well characterized internal standards is then used to develop offsets to the instrument for each level and also to periodically check the measurement drift of the instrument.

## Conclusions

Proper metrology with any type of instrument is not a trivial matter, the SEM is no different. For the precise metrology required in the manufacture of integrated circuits for submicrometer processing, an understanding of the areas that can be a problem associated with the scanning electron microscope is even more important than in any other commercial application of this instrument. The uncertainties associated with each instrument in each environment must be assessed and understood for proper metrology to be done. It has been our goal in this paper to outline some of these problems to the reader in order to put into perspective what can actually be expected from this type of instrumentation at this time. We are confident that given the necessary attention, the SEM can do the job required. As this instrument matures further in this field and research is done to improve the theoretical understanding of the physical processes going on in this instrument, the entire field of scanning electron microscopy in all its diverse applications will be furthered.

## Figures and Tables

**Table 1:** Relationship of process tolerance to the linewidth edge uncertainty.

Feature Size	10% Control	Process Tolerance (Micrometers)		LWM Edge Uncertainty
		LWM Edge Uncertainty	5% Control	
1.25	0.125	0.0625	0.0625	0.03125
1.00	0.100	0.0500	0.0500	0.02500
0.75	0.075	0.0375	0.0375	0.01875
0.50	0.050	0.0250	0.0250	0.01250
0.25	0.025	0.0125	0.0125	0.00625
0.10	0.010	0.0050	0.0050	0.00250

**Table 2:** Typical inspection instrument allocation scheme for a semiconductor processing facility.

Basic Material	Device Type	Minimum Linewidth		Measurement Instrument	
		Production	R&D	Production	R&D
**Silicon**	Large Scale Integrated Circuit	1.5 μm	1.2 μm	Optical	Optical
	High Speed Bipolar Integrated Circuit	1.0 μm	0.5 μm	Optical	SEM
	Transistor	0.8 μm	0.5 μm	SEM	SEM
**Gallium Arsenide**	Integrated Circuit	0.8 μm	0.3–0.5 μm	SEM	SEM
	Field Effect Transistor	0.3 μm	0.25–0.3 μm	SEM	SEM

**Table 3:** Comparison of some of the advantages and disadvantages afforded by the use of the scanning electron microscope for semiconductor linewidth measurement and inspection.

**SEM VS. OPTICAL MICROSCOPE**
**Comparative Advantages**
High Resolution Potential (2–20 nm)
Excellent Depth of Focus (Field)
Flexible Viewing Angles
X-Ray Characterization
Readily Interpreted Image
**Comparative Disadvantages**
High Vacuum Required
Lower Throughput
Electron Beam/Sample Interactions
Sample Charging
No Linewidth Standard Available
Expensive

**Table 4:** Comparison of the four types of electron emitters presently in use in wafer inspection instruments. Data is for 20 keV operation.

	COMPARISON OF TRADITIONAL ELECTRON EMITTERS USED IN SCANNING ELECTRON MICROSCOPY			ZR-W (100) Emitter
	Tungsten Hair Pin	Lanthanum Hexaboride	Cold Field Emitter	
Type of Emission Source	Thermionic	Thermionic	Field	Field
Temperature (K)	2650–2900	1750–2000	300	1800
Brightness (A/Cm^2^ SR)	10^4^–10^5^	10^5^–10^6^	10^7^–10^9^	10^7^–10^9^
Virtual Source Size (Angstroms)	1,000,000	200,000	50–100	50–100
Energy Spread (eV)	2–5	1–3	0.2–0.3	0.28–0.36
Vacuum (Torr)	10^−3^–10^−5^	10^−5^–10^−7^	10^−9^–10^−11^	<10^−8^

**Table 5:** Approximate Kanaya/Okayama electron range in micrometers for silicon computed using eq 2 for several accelerating voltages.

Kanaya/Okayama Electron Range in Micrometers For Silicon								
keV	1.0	1.5	2.0	5.0	10.0	15.0	20.0	30.0
μm	0.032	0.062	0.101	0.466	1.48	2.92	4.72	9.29

**Table 6:** Relationship between the pixel point resolution of a measurement system and the linewidth resolution for several magnification ranges.

LINEWIDTH MEASUREMENT RESOLUTION (512 PIXEL POINT RESOLUTION)			
Magnification	Typical Field of View	Maximum Possible Pixel Point Resolution	Maximum Possible Linewidth Resolution
10.000×	10 μm	0.02 μm	0.04 μm
50.000×	2 μm	0.004 μm	0.008 μm
100.000×	1 μm	0.002 μm	0.004 μm

**Table 7:** Data from the measurement of a nominal 0.75 μm silicide on silicon line showing measurement variation as a function of accelerating voltage and signal detection mode.

NOMINAL 0.75 MICROMETER LINEWIDTH (AVERAGE OF 40 SCANS)				
keY	SEC	SD	BSE	SD
1.5	0.916	+−0.0140	NA	NA
3.0	0.91	+−0.0092	NA	NA
5.0	0.56	+−0.0098	NA	NA
10.0	0.774	+−0.0224	0.564	+−0.0054
20.0	0.703	+−0.0125	0.556	+−0.0073
30.0	0.669	+−0.0178	0.563	+−0.0052
AVERAG	0.802		0.561	
SD	+−0.102		+ −0.004	
SD	+−0.102		+ −0.004	
		

NA=NOT APPLICABLE

SD=STANDARD DEVIATION OF THE INDICATED AVERAGE AND IS A MEASURE OF THE VARIABILITY

**Table 8:** Comparison of the total data acquisition time necessary for a typical 1 cm^2^ die, using present techniques, as a function of the instrument magnification and the acquisition frame rate.

Magnification	Field Size (μm)	Number Of Fields	Pixel Size (nm)		Overall Data Acquisition Time (hr)		
					Frame Rate (s)		
			512	1028	1.0	0.5	0.25
10,000	10	1000×1000	19.5	9.7	277.0	139.0	69.5
5,000	20	500×500	39.1	19.5	69.0	35.0	17.5
2,500	40	250×250	78.1	38.9	17.4	8.7	4.4
1,250	80	125×125	156.2	77.8	4.3	2.2	1.1
600	160	63×63	312.5	155.6	1.1	0.55	0.28

## Footnotes

1 Figures in brackets indicate literature references.
